# Lysine Depletion during Different Feeding Phases: Effects on Growth Performances and Meat Quality of Broiler Chickens

**DOI:** 10.3390/ani11061499

**Published:** 2021-05-21

**Authors:** Francesca Soglia, Marco Zampiga, Giulia Baldi, Yuwares Malila, Krittaporn V. Thanatsang, Yanee Srimarut, Nantawat Tatiyaborworntham, Onuma Unger, Annop Klamchuen, Luca Laghi, Massimiliano Petracci, Federico Sirri

**Affiliations:** 1 Department of Agricultural and Food Sciences, Alma Mater Studiorum–University of Bologna, 47521 Cesena, Italy; francesca.soglia2@unibo.it (F.S.); giulia.baldi4@unibo.it (G.B.); l.laghi@unibo.it (L.L.); m.petracci@unibo.it (M.P.); 2 Department of Agricultural and Food Sciences, Alma Mater Studiorum–University of Bologna, 40064 Ozzano dell’Emilia, Italy; marco.zampiga2@unibo.it; 3 National Center for Genetic Engineering and Biotechnology (BIOTEC), Thailand Science Park, Pathum Thani 12120, Thailand; yuwares.mal@biotec.or.th (Y.M.); krittaporn.tha@ncr.nstda.or.th (K.V.T.); yanee.sri@biotec.or.th (Y.S.); nantawat.tat@biotec.or.th (N.T.); 4 National Nanotechnology Center (NANOTEC), National Science and Technology Development Agency (NSTDA), Thailand Science Park, Pathum Thani 12120, Thailand; onuma.nanomail@gmail.com (O.U.); annop@nanotec.or.th (A.K.)

**Keywords:** lysine restriction, growth performances, meat quality, breast muscle abnormalities

## Abstract

**Simple Summary:**

In the past, many studies have been carried out to investigate the effect of lysine supplementation on broilers’ growth performances and feed efficiency. However, the knowledge concerning the reduction of the dietary content of this essential amino acid is limited and mainly restricted to the production performances of the birds. Within this context, the present study aimed at assessing the impact of lysine restriction during grower I (10–20 d) and grower I and II (10–20 and 21–34 d) feeding phases on live performances, breast meat quality traits and technological properties, as well as on the incidence and severity of abnormalities affecting the pectoral muscles (i.e., white striping, wooden breast and spaghetti meat). Lysine restriction during different feeding phases exerts negligible effects on the production performances of the broilers due to their compensatory growth. In addition, the increased anserine content following lysine depletion might have exerted a protective role against protein denaturation by buffering the acidic end-products generated during post-mortem rigor development.

**Abstract:**

The present study aimed at assessing the impact of lysine restriction performed during different feeding phases on growth performances, meat quality traits and technological properties as well as on the incidence and severity of breast muscle abnormalities. For this purpose, a total of 945 one-day-old Ross 308 male chicks was randomly divided into three experimental groups: CONT, fed a four feeding phases commercial diet, GRW I, and GRW I + II fed CONT diet with the depletion of synthetic lysine during grower I and grower I and II feeding phases, respectively. Productive performances were recorded throughout the whole rearing cycle and the incidence of breast muscle growth-related abnormalities assessed at slaughter (49 d) on 280 breasts/group. Quality traits and technological properties of breast meat were measured on a total of 54 *Pectoralis major* muscles. Lysine restriction only marginally affected the productive performances and the quality parameters of breast meat. The increased (*p* < 0.05) solubility of the protein fraction along with the remarkably higher (*p* < 0.05) anserine content found in GRW I + II suggests an increased energy requirement in the pectoral muscles belonging to lysine-restricted birds and supports the hypothesis of a reduced protein synthesis taking place within these muscles.

## 1. Introduction

Lysine (Lys) is one of the most limiting essential amino acids in commercial corn-soybean meal diets for broiler chickens [1]. Due to its relevant effect on growth performances, immune response and development of the edible skeletal muscles (especially breast, which is the most valuable portion of the chicken carcass), Lys content in broilers’ diet is accurately regulated and adjusted to ensure proper productive outcomes [2,3,4,5]. 

Lys requirements for poultry species have been updated for the last time in 1994 by the National Research Council (NRC). Lys needs have been established at 1.1% for 0 to 3 weeks old broilers and, in absolute term, this level tends to decrease as the birds grow older [6]. In general, defining the optimal amount of nutrients in broilers’ diet is complex as many of them are interdependent [6]. Indeed, many factors (including strain, sex, age and the eventual antagonism or synergistic effect of other dietary components) can affect the Lys requirement of broilers [7]. Moreover, it is worth mentioning that the nutritional requirement, along with the recommended daily intake, for this essential amino acid has surely undergone profound changes as a consequence of the selection practices carried out starting from 1950s. Indeed, the impressive growth rate and breast muscle development achieved by the modern hybrids selected for high meat yield undoubtedly imply the need for an adequate energy and nutrients supply exceeding those ones required for the past genotypes or autochthonous breeds. For this reason, the commercial broiler diets are currently formulated including a Lys level which is higher than that suggested by the NRC [6]. In addition, according to the widely known ideal protein concept, the dietary Lys concentration is crucial to define the minimum content of the other essential amino acids in the diet [8].

In the past years, studies carried out to investigate the effect of different Lys supplementations on broilers’ diet evidenced an improvement in both production performances and body composition of the birds by reducing carcass fatness and increasing meat yield [2,4,9,10,11,12,13]. In addition, the progressive enhancement in water holding capacity, as depicted by lower drip losses, together with the higher ultimate pH observed as the dietary Lys increased [14], highlights the potential role of this essential amino acid in defining the quality traits of the forthcoming meat. Despite the huge number of studies performed to evaluate the effects of Lys supplementation, the knowledge concerning the reduction of the dietary content of this essential amino acid below the recommended daily intake is limited and mainly restricted to the production performances of the birds. However, it is worth mentioning that the reduction of growth rate during some critical phases of muscle development by means of dietary Lys restriction was found to affect the overall quality of the forthcoming meat, including the occurrence of growth-related breast abnormalities [15,16], while maintaining acceptable growth performances in the whole rearing period. Therefore, the present study aimed at assessing the impact of Lys restriction during grower I (10–20 d, GRW I) and grower I and II (10–20 and 21–34 d, GRW I + II) feeding phases on growth performances, breast meat quality traits and technological properties as well as on incidence and severity of breast muscle abnormalities.

## 2. Materials and Methods

### 2.1. Animals and Housing

A total of 945 one-day-old Ross 308 male chicks were obtained from the same breeder flock and hatching session. After the vaccination procedure (coccidiosis, infectious bronchitis virus, Marek’s disease virus, Newcastle and Gumboro disease), which was carried out at the hatchery, chicks were transferred to an environmentally controlled poultry facility and then randomly allotted in 21 pens of 6 m^2^ each (7 replications/group, 45 birds/replication, approx. 8 birds/m^2^). Chopped straw (2 kg/m^2^) was utilized as litter. To reduce any environmental effect, replications were organized in randomized blocks within the poultry house. According to the legislation in force, stocking density was defined to reach a maximum of 33 kg/m^2^ [17]. Similarly, 23L:1D of artificial light was used in the first 7 d and in the last 3 d of the trial, whereas in the other days a photoperiod of 18L:6D was adopted [17]. Two circular pan feeders, able to guarantee at last 2 cm of front space/bird, and 10 nipples were provided for each pen. The environmental temperature was defined according to the age of the birds as indicated in the management guide. Birds were handled, raised and processed strictly following the European Union legislation in force [17,18,19]. The experiment was approved by the Ethical Committee of the University of Bologna (ID: 118120/2021).

### 2.2. Experimental Diets

**Figure 1:**
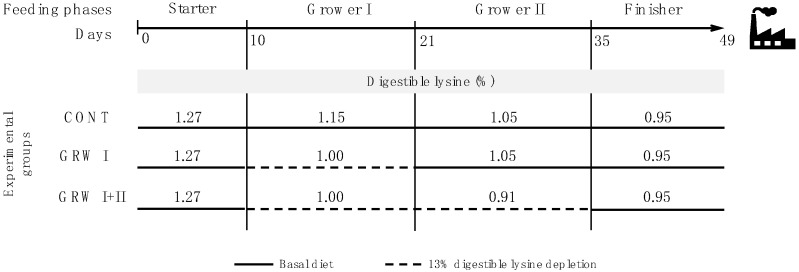
Representative diagram showing the percentages of digestible lysine (for each feeding phase) in the diets provided to broiler chickens belonging to CONT, GRW I and GRW I + II experimental groups.

The experimental diets were produced from the same commercial corn-wheat-soybean basal diet (Table 1), which presented 4 feeding phases: starter (0–9 d), grower I (10–20 d), grower II (21–34 d) and finisher (35–49 d). The basal diet was formulated to meet or slightly exceed the current nutritional recommendations for fast-growing broilers [20], with the only exception of Lys during grower I and grower II phases. In the control (CONT) diet, synthetic Lys (purity 54.6%, Evonik Industries, Hanau, Germany) was added to the aforementioned basal diet at a level of 0.0, 2.8, 2.7 and 0.0 g/ton in starter, grower I, grower II and finisher phase, respectively. Accordingly, as shown in Figure 1, the digestible Lys (dig. Lys) content of CONT diet was equal to 1.27, 1.15, 1.05 and 0.95%, respectively, for each abovementioned feeding phase, which is consistent with the current nutritional recommendations. As regards the GRW I group, synthetic Lys was added (2.7 g/ton) only in the grower II diet (21–34 d), thereby resulting in a sub-optimal Lys level during the grower I phase (dig. Lys = 1.00%). On the other hand, no synthetic Lys was supplemented in the GRW I + II diet, thus determining reduced Lys levels during both the grower I and grower II phases (dig. Lys = 1.00 and 0.91%, respectively). All diets were provided in a mash form and, as well as water, administered for ad-libitum consumption. Digestible amino acid values were calculated by multiplying digestibility coefficients [21] to the analyzed total amino acid content of each ingredient. 

### 2.3. Productive Performances

At the end of each feeding phase (9, 20, 34 and 49 d), all birds were weighed, and feed consumption was determined on a pen basis. Productive performance parameters such as body weight (BW), daily weight gain (DWG), daily feed intake (DFI) and feed conversion rate (FCR), were calculated accordingly. Dead or culled animals were daily recorded and weighed to determine the mortality percentage and to correct performance data. At 49 d, all birds were processed in a commercial slaughterhouse. Birds were stunned by means of an electrified water-bath (200 to 220 mA, 1500 Hz) according to the current legislation [18]. 

### 2.4. Incidence of Growth-Related Breast Meat Abnormalities

The incidence and severity of muscular abnormalities including White Striping (WS), Wooden Breast (WB) and Spaghetti Meat (SM) were evaluated on a total of 840 *Pectoralis major* muscles (280/experimental group), which were randomly collected 24 h post-mortem. In detail, a 3 point-scale evaluation system (NORM: no lesions; MOD: moderate lesions; SEV: severe lesions) was used to assess the presence of each defect and classify the *P. major* according to the criteria proposed in previous studies [22,23,24]. In order to avoid misleading results, all breasts were scored by the same well-trained operators in analogous environmental conditions.

### 2.5. Meat Quality

A total of 54 *P. major* muscles (18/experimental group) were selected 24 h post-mortem from carcasses having a BW corresponding to the average value of each group. In addition, in order to avoid any interference ascribable to the presence of muscular defects/myopathies, muscles exhibiting color defects and/or macroscopic features associated to WS, WB and SM conditions were not considered. After being selected, 12 *P. major* muscles/experimental group were transported under refrigerated conditions to the laboratories of the Department of Agricultural and Food Sciences of the University of Bologna to assess the main quality traits (ultimate pH and color) and technological properties (drip and cooking loss) of meat as well as assess the content of histidine-containing dipeptides, and metabolites belonging to energy-generating pathways through ^1^H-NMR spectroscopy. Furthermore, 6 *P. major* muscles/experimental group were frozen at −80 °C and delivered to the Food Biotechnology Research Team, National Center for Genetic Engineering and Biotechnology (Pathum Thani, Thailand) in order to further study the protein fraction (amino acid profile and protein solubility) and its oxidative stability.

#### 2.5.1. Quality Traits and Technological Properties

Following the removal of superficial fat and connective tissue, meat color [lightness (L*), redness (a*) and yellowness (b*)] was measured in triplicate on the bone side surface of each muscle by a reflectance colorimeter (Minolta Chroma Meter CR-400, Minolta Italia S.p.A., Milan, Italy) using illuminant source C. Ultimate pH was determined according to the iodoacetate method proposed by Jeacocke [25]. As for the Water Holding Capacity (WHC) of the meat, a parallelepiped meat cut (8 × 4 × 3 cm, weighing about 75 g) was excised from the cranial portion of each muscle, stored at 4 ± 1 °C for 72 h in plastic boxes over sieved plastic racks and drip loss was calculated as the percentage of weight lost during storage. Then, each sample was individually packaged under vacuum and cooked in a water bath at 80 °C until reaching the same temperature in its inner core (to avoid the development of a temperature gradient within the samples). After allowing the samples to equilibrate to room temperature, cooking loss was calculated as the percentage of weight lost during cooking.

#### 2.5.2. Amino Acid Profile

Amino acid profile of the breast meat was assessed using a gas chromatography-mass spectrometry (GC-MS) technique. Prior to analysis, 40 mg (in triplicates) of raw meat were hydrolyzed (at 110 °C for 18 h) in 5 mL of 6 N HCl. After adjusting the pH of each hydrolysates to a pH range from 1.0 to 2.0, 25 µL of each sample and 50 μL of 0.2 mM norleucine (as internal standard) were transferred into a GC vial. The samples were then dried and derivatized (at 100 °C for 4 h) through incubation with 50 μL of N-tert-butyldimethylsilyl-N-methyltrifluoroacetamide containing 1% tert-butyldimethyl chlorosilane and 50 µL acetonitrile. The derivatized samples were subsequently analyzed using a 7890A GC/5975C MS (Agilent Technologies, Santa Clara, CA, USA). Samples were injected using a split mode with helium as carrier gas and separated through a DB-5 column (30 m × 0.25 mm initial diameter × 0.1 µm thickness, Agilent Technologies). The oven temperature was programmed to range from 170 °C (5-min hold) to 200 °C at a rate of 4 °C/min, followed by 3 min hold at 200 °C and to 285 °C at a rate of 4 °C/min. The MS condition was set as follows: transfer line 300 °C, ion source 230 °C, and electron impact ionization 70 eV. The scan range was 35 to 800 *m*/*z*. The scan mode was used for data acquisition. Standard mixtures of amino acids with known concentrations were prepared to generate calibration curves where peak area of each amino acid was plotted against the known concentrations in the standard mixture. The amino acid content was then calculated and expressed as g/100 g of meat sample. 

#### 2.5.3. Protein Solubility

Protein solubility was determined following the procedure described by Mudalal et al. [26] with slight modifications. A 400 mg meat sample was homogenized by an Ultra-Turrax T25 (IKA^®^-Werke GmbH and Co., Staufen, Germany) (in ice, at 13,000 rpm for 2 min) in 5 mL of a 1.1 M KI, 0.1 M potassium phosphate buffer (pH 7.2). The homogenate was centrifuged at 10,000× *g* at 4 °C for 10 min. The volume and weight of the resulting supernatant and pellet were recorded. Protein concentration in the supernatant was assessed by using bicinchoninic acid assay with bovine serum albumin (BSA) as a standard and the 1.1 M KI in 0.1 M potassium phosphate (pH 7.2) buffer as blank and expressed as mg/100 mg of total protein. Total protein of each meat sample was determined using Kjeldahl method according to the Association of Official Analytical Chemists [27].

#### 2.5.4. Protein Carbonyls

Carbonyl concentration was determined using a Protein Carbonyl Content Assay Kit (Sigma-Aldrich, St. Louis, MO, USA). Proteins were extracted as previously described by Soglia et al. [28] with slight modifications. After extraction with 1.1 M KI, 0.1 M potassium phosphate buffer (pH 7.2) (see Section 2.5.3), the proteins in the supernatant (approximately 2.0 mg) were precipitated by centrifugation (3500× *g* for 2 min) following the addition of 1 mL of ice-cold acetone. The resultant pellet was re-suspended in 400 µL of 5% (*w*/*v*) sodium dodecyl sulfate (SDS) and incubated at 100 °C for 10 min with a constant shaking (1500 rpm). Then, following the manufacturer’s procedure, 100 µL of 2,4-dinitrophenylhydrazine (DNPH) reagent were added to an aliquot (200 µL) of SDS-solubilized proteins and the samples were subsequently incubated at room temperature for 10 min. Following 3 cycles of washing with ice-cold acetone to precipitate the protein and remove free DNPH, the protein pellet was re-solubilized in 150 µL of 6 M guanidine hydrochloride at 60 °C with a constant shaking at 1200 rpm for 30 min. The protein concentration was determined by a bicinchoninic acid assay using BSA as a standard reference and carbonyl content (expressed as nmol/mg protein) was calculated using a millimolar extinction coefficient of 22 mM^−1^ cm^−1^ after reading the absorbance at 375 nm.

#### 2.5.5. Sulfhydryl Content

Total sulfhydryl (TSH) and surface sulfhydryl (SSH) groups were determined based on the methods described by Benjakul et al. [29] and Visessanguan et al. [30], respectively, with slight modifications. The protein concentration in the supernatant obtained after centrifuging the samples with 1.1 M KI, 0.1 M potassium phosphate buffer (pH 7.2) (see Section 2.5.3) was adjusted to 4 mg/mL with deionized water. To determine TSH, an aliquot (500 µL) was mixed with 1.5 mL of working reagent, containing 0.2 M Tris-HCl, 8 M urea, 2% (*w*/*v*) SDS and 10 mM EDTA buffer (pH 6.8), followed by the addition of 200 µL of 0.1% (*w*/*v*) 5,5′-dithio-bis-(2-nitrobenzoic acid) (DTNB) in 0.2 M Tris-HCl (pH 8.0). As per SSH, the sample (500 µL) was treated similarly but the working reagent was in absence of 8 M urea. After incubating the mixture for 25 min at 40 °C or for 25 min (TSH) at 5 °C (SSH) and reading the absorbance at 412 nm, the amount of TSH and SSH was calculated by using a molar extinction coefficient of 13,600 M^−1^ cm^−1^ and 14,150 M^−1^ cm^−1^, respectively. Both values were finally expressed as µmol/mg meat.

#### 2.5.6. Histidine-Containing Dipeptides and Metabolites 

^1^H-NMR spectroscopy was performed on a 500 mg meat sample homogenized by Ultra-Turrax (11,000 rpm, 20 s) in 3 mL of distilled water following the procedure previously described by Soglia et al. [31]. An aliquot (1 mL) of homogenate was centrifuged (10 min, 18,000× *g* at 4 °C) and 700 µL of the supernatant were added with 800 µL Chloroform. Samples were mixed by vortex (2 min), centrifuged (2 min, 18,000× *g* at 4 °C) and 500 µL of the supernatant were mixed with 200 µL of 1 M potassium phosphate buffer (containing 2 mM sodium azide; pH 7.0) in D_2_O and 10 mM 3-(Trimethylsilyl) propionic-2,2,3,3-d4 acid sodium salt (TSP). After centrifuging (10 min, 18,000× *g* at 4 °C) the samples, 700 µL of supernatant were transferred to NMR tube for analysis. ^1^H-NMR spectra were acquired using the cpmgpr1d pulse sequence with suppression of the solvent signal and the following parameters were set: size of fid: 32 k, number of scans: 16, number of dummy scans: 16, spectral width: 12 ppm, acquisition time: 2.28 s, delay d1: 5 s. NMR spectra were processed, adjusted and quantified with Topspin 3.1 and R software while elucidation and identification of the compounds was done with the help of the HMDB database (http://www.hmdb.ca/, accessed on 28 April 2021), Chenomx software and literature references. For quantification and calibration of the spectrum, the TSP was used (δ 0.00).

#### 2.5.7. Buffering Capacity

Buffering capacity was assessed following the procedure proposed by Matarneh et al. [32] with slight modifications [33]. Briefly, 2.5 g of meat were homogenized with an Ultra-Turrax T-25 (IKA-Werke, Germany) in 25 mL of Jeacocke solution (5 mM sodium iodoacetate and 150 mM KCl, pH 7.0) [25]. After allowing the samples to equilibrate to 25 °C, the homogenate was transferred into a beaker and the initial pH (pHi) measured while stirring with a pH glass electrode (Jenway, Staffordshire, UK). Then, titration was performed up to pH 7.0 using 0.5 M NaOH and buffering capacity calculated as follows: 

Buffering capacity = ΔB/ΔpH, where ΔB is the increment of base expressed as μmol NaOH/g of tissue and ΔpH is the corresponding pH variation after the addition of NaOH.

### 2.6. Statistical Analyses

Data were analyzed by one-way ANOVA considering the feeding phase (grower I or grower I and II) in which Lys restriction has been performed as the main effect. Means were subsequently separated through the parametric Tukey-HSD test. In addition, in order to further investigate the effect of Lys restriction, planned orthogonal contrasts were performed to compare the findings obtained within the control group (CONT) with those found in the treated ones (GRW I and GRW I + II). All statistical differences were considered significant at a level of *p* ≤ 0.05. All statistical analyses were carried out by using Statistica software (StatSoft Italy srl, Vigonza, Italy). Pen was considered as the experimental unit for productive performance data and, prior to analysis, mortality data were submitted to arcsine transformation. For all the other parameters, the experimental unit was the breast sample. 

## 3. Results

### 3.1. Productive Performances

Table 2 shows the results of productive performances. At placement, chicks assigned to CONT, GRW I and GRW I + II groups showed similar BW. As expected, no significant difference was observed among the groups during the starter phase (0–9 d). From 10 to 20 d, both GRW I and GRW I + II groups showed lower DFI compared to CONT (77.7 and 76.4 vs. 81.2 g/bird/d, respectively; *p* < 0.01). On the other hand, DWG and BW tended to be lower in GRW I and GRW I + II groups in respect to CONT (50.3 and 49.9 vs. 52.0 g/bird/d, *p* = 0.07; and 772 and 764 vs. 789 g, *p* = 0.10, respectively). Considering the orthogonal contrasts, such differences achieved the statistical significant threshold, thereby confirming that Lys-depleted groups were characterized by lower BW and DWG compared to the CONT one. Consistently, FCR was not significantly affected by the dietary treatment. From 21 to 34 d, Lys depletion determined lower DFI in GRW I + II group (145.1 vs. 152.9 g/bird/d, respectively, for GRW I + II and CONT; *p* < 0.01). Conversely, birds belonging to the GRW I group showed lower DFI than the CONT ones (146.5 vs. 152.9 g/bird/d, respectively; *p* < 0.01), although both these groups received the same diet during this feeding phase. Moreover, GRW I + II birds exhibited lower BW than CONT ones (1890 vs. 2014 g, respectively; *p* < 0.05), whereas GRW I group showed intermediate values. Neither DWG nor FCR were significantly modified, even if a trend for reduced DWG emerged for Lys-depleted groups. Furthermore, no significant difference among the groups was observed during the finisher phase (35–49 d), in which all the experimental groups received the CONT diet. In the overall period of the trial (0–49 d), CONT, GRW I and GRW I + II groups presented comparable growth performances, although lower DFI was observed for Lys-depleted groups according to the planned orthogonal contrasts. Finally, mortality did not present significant variations among the groups either considering each feeding phase or the overall period of the trial. 

### 3.2. Incidence of Breast Meat Abnormalities

**Figure 2:**
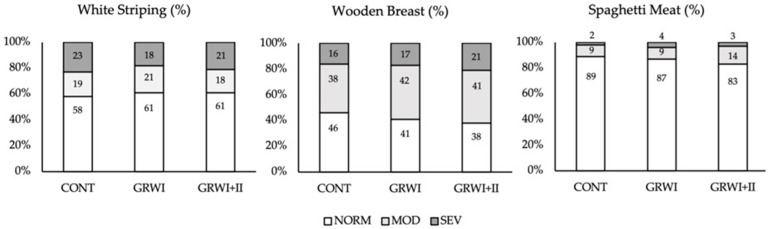
Incidence (%) and severity (moderate—MOD or severe—SEV) of growth-related abnormalities affecting the *Pectoralis major* muscles in broilers belonging to CONT, fed a four feeding phases commercial diet, GRW I and GRW I + II fed CONT diet with the depletion of synthetic lysine during grower I and grower I and grower II feeding phases.

The incidence and severity of WS, WB and SM abnormalities are reported in Figure 2. Lys depletion either during the grower I (10–20 d) or grower I and II phases (10–20 d and 21–34 d) did not significantly affect the occurrence of WS defect at 49 d. Similarly, the groups presented comparable incidence and severity of WB abnormality. At last, the overall occurrence of SM abnormality ranged from 11 to 17%, with only minimal differences among the experimental groups. 

### 3.3. Meat Quality

#### 3.3.1. Quality Traits and Technological Properties

The main quality traits and technological properties of the *P. major* muscles were similar regardless of the experimental group (Table 3) with the only exception of yellowness (b*) which was found to be higher by planned orthogonal contrasts carried out to compare CONT group and its treated counterparts (GRW I and GRW I + II; LSM: 3.49 vs. 2.04 and 2.61; *p* < 0.05). Overall, no differences in ultimate pH, color parameters and WHC were observed either during refrigerated storage up to 96 h (drip loss) or following cooking (cooking loss). 

#### 3.3.2. Protein Oxidation and Functionality

The oxidative stability of meat proteins was not affected by Lys depletion carried out during different growing phases (GRW I or GRW I + II). Indeed, both the carbonylation level and the content of TSH and SSH were similar among the groups (*p* > 0.05) (Table 4). On the other hand, Lys restriction carried out on both grower I and II feeding phases remarkably affected the protein’s solubility. Indeed, if compared to the values observed in CONT and GRW I, a significantly higher solubility of the protein fraction was found in GRW I + II (74.1 and 73.4 vs. 77.2 mg/100 mg of total protein; *p* < 0.05).

#### 3.3.3. Amino Acid Profile

Overall, the amino acid profile of the breast meat was modified by Lys depletion implemented during different feeding phases (Table 5). 

In detail, through planned orthogonal contrasts, a significantly lower alanine, glycine, proline and aspartate content was found within the *P. major* muscles belonging to Lys-depleted groups if compared to the CONT. In addition, Lys restriction implemented during both grower I and II feeding phases (GRW I + II) resulted in increased isoleucine and histidine contents (*p* < 0.001 and *p* < 0.01, respectively).

#### 3.3.4. Histidine-Containing Dipeptides and Metabolites

The findings concerning the concentration of histidine-containing dipeptides and metabolites assessed through ^1^H-NMR spectroscopy are reported in Table 6. 

As for histidine-containing dipeptides, Lys depletion resulted in a higher anserine content within the pectoral muscle belonging to treated groups (GRW I and GRW I + II) in comparison with CONT (LSM: 429.4 and 442.3 vs. 369.7 mg/100 g of meat; *p* < 0.05), as detected by planned orthogonal contrasts. On the other hand, the concentration of the main metabolites involved in energy-generating pathways was found to be similar (*p* > 0.05) among the experimental groups. However, by means of planned orthogonal contrasts, a tendency was found for lactate and hypoxanthine which were found to be higher in Lys-depleted groups in respect to the CONT (LSM: 692.9 and 677.5 vs. 611.1 mg/100 g of meat, *p* = 0.06; LSM: 27.6 and 27.1 vs. 23.4 mg/100 g of meat, *p* = 0.09, respectively).

#### 3.3.5. Buffering Capacity

The buffering capacity of the *P. major* muscle was remarkably affected by Lys depletion performed during different feeding phases (Table 7). 

Indeed, Lys restriction carried out during both the grower I and II feeding phases (GRW I + II group) resulted in a significantly higher ability of the pectoral muscle to buffer hydrogen ions within the pH range 6.6–7.0 (51.6, 58.0 and 55.2 µmol H^+^·pH^−1^·g^−1^). 

## 4. Discussion

Overall, Lys depletion during grower I and II feeding phases only marginally affected the productive performances and the quality parameters of the breast meat. As concerns growth performances, the main effect of Lys restriction during grower I phase (10–20 d) was observed on DFI which was significantly lower in broilers receiving diets with suboptimal Lys levels. The orthogonal contrasts revealed that DWG and BW in Lys-depleted groups at 20 d were lower than those of CONT, although only a tendency was observed when considering each individual group. It is generally thought that broiler chickens tend to compensate for differences in nutrient density by increasing feed intake in order to meet their amino acid or energy requirements [16]. However, De Cesare et al. [34] reported that chickens fed diets with adequate or reduced (−7%) crude protein content presented comparable feed consumption, thereby indicating that they did not adjust feed intake according to the dietary crude protein content. Meloche et al. [16], finding no differences in feed intake when diets with different dig. Lys content were fed from 12 to 18 d, concluded that broilers are less sensitive to variations in dietary Lys content. Intriguingly, GRW I and GRW I + II groups showed lower feed intake than CONT birds. Previous findings indicate that reduced dig. Lys diets can have an appetite-depressant effect in young broilers [35], which is mediated by variations in the plasma levels of Lys [36,37]. On the other hand, the reduction of Lys concentration could have affected the ratio between Lys and other dietary components, such as the metabolizable energy, which might have played a role in altering voluntary feed consumption. Further insights are thus necessary to define the relationship between dietary Lys depletion and reduced feed intake in broiler chickens.

Extending the Lys depletion even to the grower II phase determined a significant reduction of BW and DFI in comparison to the CONT, whereas FCR was not significantly affected by the dietary treatments. Meloche et al. [16] reported that the growth pattern of broilers from 12 to 18 d was altered by the reduction of dig. Lys to 75% of the recommended density (dig. Lys = 1.17%). On the other hand, reducing the dig. Lys levels to 85% of the recommended dose had no effect on body weight either from 12 to 18 d or 12 to 26 d [16]. In the present study, the reduction of dig. Lys during grower I (10–20 d) was equal to 13% (i.e., from 1.15 to 1.00%), which could explain the lack of differences in growth performances among the experimental groups. However, protracting the reduction of Lys density also in the grower II phase significantly lowered body weight, likely due to a cumulative effect on feed intake depression. Such variations of growth trajectories observed in the grower I and II phases were not statistically relevant when considering the overall period of trial, with the only exception of feed intake that was lower in GRW I + II in comparison to the control. Overall, these results indicated that broilers are able to recover from reductions in Lys density during certain phases of the rearing cycle, which is consistent with previous findings [16]. 

The dig. Lys depletion tested in the present study did not significantly affect the incidence and severity of WS, WB, and SM abnormalities. Meloche et al. [16] reported that broilers receiving diets formulated at 75% of the recommended dig. Lys concentrations from 12 to 26 d of age showed a lower occurrence of severe cases of WB and WS in comparison to the control group at 48 d. These authors associated such reduction to the lower breast muscle weight and yield observed in that group which, in turn, has been related to a limited intake of dietary Lys. However, an 85% reduction of dig. Lys concentration during 12 to 18 d or 12 to 26 d of age had no significant effect on the occurrence of severe cases of both WS and WB. Taken together, these findings suggest that the effects of dietary Lys depletion on the occurrence of growth-related abnormalities depends on both the extent of its reduction and to the duration of such reduction in respect to the overall rearing period. Likely, the Lys depletion tested in the present study (i.e., −13%) was not sufficient to significantly mitigate the incidence levels of WS, WB, and SM at 49 d. 

Even though in previous studies the supplementation of Lys was associated to an improvement in meat quality parameters (e.g., higher ultimate pH leading to an enhanced WHC) [14], this trend was not observed when Lys is depleted [16]. Lys restriction performed during different feeding phases exerted some effects on meat quality parameters, although its consequences were not necessarily substantial neither adverse. These outcomes may likely be ascribed to the Lys depletion level (i.e., −13%) performed within this study which was not such as to affect the main quality traits of the forthcoming meat likely due to the compensatory growth which allowed to obtain an analogous development of the skeletal muscles. Overall, these findings are in agreement with those obtained in a recent study [16] in which meat quality traits assessed at slaughter and 24 h post-mortem were not affected by a 15% reduction of dig. Lys in the diet. On the other hand, supplementing the diet with increasing dig. Lys concentrations was previously associated with overall improved meat quality parameters [10,14]. In detail, a remarkable reduction (−20%) in the amount of liquid lost during refrigerated storage intrinsically linked to a steady increase in ultimate pH was found as the dig. Lys in the diet increased [14].

The most interesting result is the increased protein solubility found in the pectoral muscles belonging to birds fed low Lys diets, with special reference to GRW I + II group. Nevertheless, this increased functionality of the protein fraction was not mirrored by an improvement in WHC of the meat itself and may be likely ascribed to a different acidification pattern occurring during post-mortem time. Indeed, pH decline taking place during early post-mortem exerts a major role in defining the functionality of the meat proteins [38,39]. This is particularly evident when the consequences of a fast acidification taking place early post-mortem are considered [40,41,42,43]. Under these circumstances, indeed, a partial protein denaturation occurs and is hence associated with an impaired protein functionality [40,44,45]. Considering the above, the significantly higher protein solubility observed within the pectoral muscles belonging to Lys-restricted groups, with special reference to GRW I + II, might be attributed to their anserine content. Indeed, aside from acting as a scavenger against free radicals [46,47,48], this histidine-containing dipeptide has also been demonstrated to play a role in buffering the acidic end-products of the energetic metabolism either in vivo or during post-mortem time [49]. In agreement with that, pectoral muscles belonging to GRW I + II group exhibited a significantly higher ability to buffer the hydrogen ions within the pH range from 6.6 to 7.0, which approximates the pK_a_ value of anserine [50]. Thus, considering the increased protein solubility/functionality together with the higher buffering capacity observed in the *P. major* belonging to Lys-restricted groups, a protective role of anserine against protein denaturation induced by muscular acidification may be reasonably hypothesized. Indeed, by buffering hydrogen ions generated during early post-mortem time, anserine may have limited the rate of muscular acidification, thus protecting the polypeptide chains from denaturation and, consequently, leading to a higher functionality (as depicted by the higher solubility) of the protein fraction itself. Furthermore, in spite of the higher lactate content measured in the pectoral muscles belonging to Lys-restricted groups, the absence of significant differences in ultimate pH may be once more likely ascribed to the higher anserine and histidine content found in the aforementioned groups which may have counteracted muscles’ acidification. 

As for the amino acid profile of the breast meat, the remarkable reduction in aspartate and alanine observed within Lys-depleted groups may be associated with a lower Lys content within these muscles, although in the present study no significant differences among the groups were found concerning this amino acid. In addition, as a higher amount of proline was previously associated to an enhanced protein synthesis [51], the reduced content of this amino acid found in Lys-restricted groups may suggest an inhibited protein anabolism. Accordingly, a previous study highlighted a change in protein metabolism, mainly catabolism, when a reduction of dietary Lys was implemented in broiler chickens [52]. In addition, together with hydroxyproline, glycine and proline account for more than 50% of the total amino acids composing collagen [53]. Thus, considering their biological function, the remarkably lower amounts of these amino acids observed within the muscles belonging to Lys-restricted groups (GRW I and GRW I + II) may be associated to a reduced protein synthesis in general, and to a decreased collagen synthesis, in particular. In addition, the higher histidine level found in GRW I + II corroborates the hypothesis of a more intense anserine biosynthesis taking place within these muscles. Overall, the increased hypoxanthine content coupled with the slightly reduced inosine level observed in broilers fed low Lys diets suggests an altered energy metabolism within these muscles. This hypothesis is further corroborated by the findings obtained by Watanabe et al. [52], who speculated an increased ATP synthesis as a response towards dietary Lys restriction. This suggests an increased energy requirement in the pectoral muscles belonging to Lys-restricted birds and further supports the above-mentioned hypothesis of a reduced protein synthesis taking place within these muscles as it is generally held that protein metabolism can also be affected by the energetic status of the muscle itself [54]. 

## 5. Conclusions

Overall, the outcomes evidenced that the Lys restriction tested in this study (−13%) during the grower I and II feeding phases exerts negligible effects on the productive performances of the broilers due to their compensatory growth, thus confirming their ability to fully recover from reductions in Lys density during certain phases of the rearing cycle. Lys depletion, at least at the levels tested herein, did not significantly modify the occurrence of growth-related breast meat abnormalities. However, the increase in anserine content following Lys depletion might have exerted a protective role against protein denaturation by buffering the acidic end-products generated during early post-mortem time. Lys depletion may represent a promising strategy to modulate post-mortem acidification ultimately contributing to define the quality of the forthcoming meat. In addition, the restriction of the Lys content may represent a profitable and effective strategy to reduce feeding costs and improve the sustainability of the production system without affecting both production performances and meat quality. Indeed, it is generally held that reducing the concentration of some dietary components may result in a lower nitrogen and phosphorous excretion thus improving the environmental sustainability of the production system. 

## Figures and Tables

**Table 1:** Composition of the diet in each feeding phase.

	Starter 0–9 d	Grower I 10–20 d	Grower II 21–34 d	Finisher 35–49 d
Ingredients, g/100 g				
Corn	42.3	45.6	39.7	30.0
Wheat	12.0	12.0	17.5	30.0
Sorghum	0.00	0.00	5.01	5.00
Vegetable oil	1.61	2.31	2.48	2.59
Soybean meal 48%	17.7	20.0	13.3	6.06
Full-fat soybean	5.00	7.53	10.0	12.5
High-protein soybean meal	5.00	0.00	0.00	0.00
Sunflower	3.00	3.01	4.01	5.00
Pea	3.00	3.01	5.01	6.00
Corn gluten	3.00	0.00	0.00	0.00
Lysine	0.67	0.23	0.22	0.50
DL-Methionine	0.27	0.31	0.29	0.25
L-Threonine	0.18	0.17	0.12	0.11
Choline chloride	0.10	0.10	0.05	0.05
Calcium carbonate	0.40	0.54	0.66	0.87
Dicalcium phosphate	1.10	0.57	0.40	0.23
Sodium chloride	0.36	0.32	0.28	0.19
Sodium bicarbonate	0.00	0.00	0.10	0.19
Vitamin premix ^1^	0.50	0.45	0.36	0.21
Phytase	0.05	0.05	0.05	0.05
Xylanase	0.05	0.05	0.05	0.05
Calculated composition, %				
Dry matter *	88.3	88.1	88.3	88.4
Crude protein *	22.2	20.0	18.7	17.5
Total lipid *	4.68	5.85	6.38	6.80
Crude fiber *	2.91	2.96	2.97	3.10
Ash	4.82	4.37	4.14	4.00
Lysine (digestible)	1.27	1.15	1.05	0.95
Met. + Cyst. (digestible)	0.92	0.84	0.79	0.72
Arginine (digestible)	1.34	1.25	1.15	1.03
Threonine (digestible)	0.85	0.77	0.68	0.62
Ca (total)	0.69	0.59	0.57	0.56
P (total)	0.59	0.49	0.44	0.40
AME ^2^, kcal/kg	3000	3078	3153	3200

^1^ Provided the following per kg of diet: vitamin A (retinyl acetate), 13,000 IU; vitamin D3 (cholecalciferol), 4000 IU; vitamin E (DL-α-tocopheryl acetate), 80 IU; vitamin K (menadione sodium bisulfite), 3 mg; riboflavin, 6.0 mg; pantothenic acid, 6.0 mg; niacin, 20 mg; pyridoxine, 2 mg; folic acid, 0.5 mg; biotin, 0.10 mg; thiamine, 2.5 mg; vitamin B12 20 μg; Mn, 100 mg; Zn, 85 mg; Fe, 30 mg; Cu, 10 mg; I, 1.5 mg; Se, 0.2 mg; ethoxyquin, 100 mg. ^2^ Apparent Metabolizable Energy. * Analyzed values.

**Table 2:** Productive performance of CONT, GRW I and GRW I + II groups in each feeding phase and in the overall period of the trial.

		Experimental Group			sem	*p*-Value	
		CONT	GRW I	GRW I + II		Analysis of Variance ^1^	Planned Contrast ^2^
n. replicates		7	7	7			
*0–9 d*							
Chick body weight (g/bird)		43.3	43.2	43.1	0.10	ns	ns
Body weight (g/bird)		215	218	214	1.03	ns	ns
Daily weight gain (g/bird/d) ^#^		19.1	19.5	19.0	0.11	ns	ns
Daily feed intake (g/bird/d) ^#^		24.0	24.7	24.1	0.14	0.06	ns
Feed conversion ratio ^#^		1.259	1.269	1.269	0.01	ns	ns
Mortality (%)		0.00	0.00	0.00	-	-	-
*10–20 d*							
Body weight (g/bird)		789	772	764	4.82	0.10	*
Daily weight gain (g/bird/d) ^#^		52.0	50.3	49.9	0.40	0.07	*
Daily feed intake (g/bird/d) ^#^		81.2 a	77.7 b	76.4 b	0.64	**	**
Feed conversion ratio ^#^		1.563	1.545	1.533	0.01	ns	ns
Mortality (%)		0.32	0.00	0.32	0.01	ns	ns
*21–34 d*							
Body weight (g/bird)		2014 a	1953 ab	1890 b	21.5	*	*
Daily weight gain (g/bird/d) ^#^		87.5	84.3	80.4	1.45	ns	0.09
Daily feed intake (g/bird/d) ^#^		152.9 a	146.5 b	145.2 b	1.16	**	**
Feed conversion ratio ^#^		1.749	1750	1.809	0.02	ns	ns
Mortality (%)		0.32	0.00	0.00	0.01	ns	ns
*35–49 d*							
Body weight (g/bird)		3601	3519	3494	28.6	ns	ns
Daily weight gain (g/bird/d) ^#^		105.8	104.2	106.7	1.63	ns	ns
Daily feed intake (g/bird/d) ^#^		230.5	228.3	227.0	1.56	ns	ns
Feed conversion ratio ^#^		2.184	2.195	2.139	0.02	ns	ns
Mortality (%)		0.00	0.32	0.64	0.01	ns	ns
*0–49 d*							
Body weight (g/bird)		3601	3519	3494	28.6	ns	ns
Daily weight gain (g/bird/d) ^#^		72.6	70.9	70.4	0.58	ns	ns
Daily feed intake (g/bird/d) ^#^		136.7	133.7	132.4	0.78	0.06	*
Feed conversion ratio ^#^		1.886	1.889	1.886	0.01	ns	ns
Mortality (%)		0.64	0.32	0.96	0.02	ns	ns

^1^ One-way ANOVA considering lysine restriction implemented during different feeding phases as a main effect. ^2^ Planned orthogonal contrast: Control (CONT) vs. Treated (GRW I and GRW I + II) groups. * = *p* < 0.05; ** = *p* < 0.01; ns = not significant; ^#^ corrected for mortality; a,b = mean values followed by different letters significantly differ among the groups (*p* < 0.05).

**Table 3:** Quality traits and technological properties of breast meat from broiler chickens fed diets low in lysine at different growing phases: grower I (GRW I) or grower I and II (GRW I + II). *n* = 12 samples/group; sem = standard error of mean.

	Experimental Group			sem	*p*-Value	
	CONT	GRW I	GRW I + II		Analysis of Variance ^1^	Planned Contrast ^2^
Weight (g)	303.35	259.37	267.43	5.54	ns	ns
Ultimate pH	5.71	5.66	5.67	0.02	ns	ns
Lightness—L *	58.30	57.98	57.31	0.45	ns	ns
Redness—a *	1.78	1.84	1.81	0.10	ns	ns
Yellowness—b *	2.76	2.30	2.36	0.18	ns	*
Drip loss 24 h (%)	1.83	1.82	1.68	0.05	ns	ns
Drip loss 96 h (%)	2.70	2.79	2.48	0.10	ns	ns
Cooking loss (%)	22.94	22.90	22.47	0.31	ns	ns

^1^ One-way ANOVA considering lysine restriction implemented during different feeding phases as a main effect. ^2^ Planned orthogonal contrast: Control (CONT) vs. Treated (GRW I and GRW I + II) groups. * = *p* < 0.05; ns = not significant.

**Table 4:** Oxidative stability and functionality of the proteins composing the *Pectoralis major* muscles from broiler chickens fed diets low in lysine during different feeding phases: grower I (GRW I) or grower I and II (GRW I + II). *n* = 3 samples/group; sem = standard error of mean.

	Experimental Group			sem	*p*-Value	
	CONT	GRW I	GRW I + II		Analysis of Variance ^1^	Planned Contrast ^2^
Carbonyls (nmol/mg of protein)	2.94	2.93	3.20	0.19	ns	ns
Protein solubility (mg/100 mg total protein)	74.1 b	73.4 b	77.2 a	1.61	*	*
TSH (μmol/mg meat)	6.52	6.23	6.65	0.06	ns	ns
SSH (μmol/mg meat)	5.90	6.01	5.84	0.08	ns	ns

^1^ One-way ANOVA considering lysine restriction implemented during different feeding phases as a main effect. ^2^ Planned orthogonal contrast: Control (CONT) vs. Treated (GRW I and GRW I + II) groups. * = *p* < 0.05; ns = not significant. a,b = mean values followed by different letters significantly differ among the groups (*p* < 0.05).

**Table 5:** Amino acid profile of the *Pectoralis major* muscles from broiler chickens fed diets low in lysine during different feeding phases: grower I (GRW I) or grower I and II (GRW I + II). Data are expressed as g/100 g of meat; *n* = 6 samples/group; sem = standard error of mean.

	Experimental Group			sem	*p*-Value	
	CONT	GRW I	GRW I + II		Analysis of Variance ^1^	Planned Contrast ^2^
Alanine	1.33 a	1.22 b	1.30 ab	0.02	*	*
Glycine	1.00 a	0.84 b	0.86 b	0.02	**	***
Valine	0.72	0.65	0.69	0.03	ns	ns
Leucine	1.88	1.83	1.91	0.02	ns	ns
Isoleucine	1.05 b	1.03 b	1.12 a	0.01	***	ns
Proline	1.25 a	0.83 b	1.00 ab	0.06	**	**
Methionine	0.47	0.47	0.51	0.01	ns	ns
Serine	0.71	0.67	0.72	0.02	ns	ns
Threonine	0.96	0.92	0.97	0.01	ns	ns
Phenylalanine	0.71	0.69	0.78	0.02	ns	ns
Aspartate	2.13 a	1.97 b	2.05 ab	0.02	*	**
Glutamate	3.24	3.08	3.21	0.04	ns	ns
Lysine	2.20	2.03	1.93	0.07	ns	ns
Arginine	1.45	1.35	1.53	0.04	ns	ns
Histidine	1.14 ab	1.10 b	1.19 a	0.01	**	ns
Tyrosine	0.42	0.38	0.47	0.03	ns	ns

^1^ One-way ANOVA considering lysine restriction implemented during different feeding phases as a main effect. ^2^ Planned orthogonal contrast: Control (CONT) vs. Treated (GRW I and GRW I + II) groups. * = *p* < 0.05; ** = *p* < 0.01; *** = *p* < 0.001; ns = not significant. a,b = mean values followed by different letters significantly differ among the groups (*p* < 0.05).

**Table 6:** Histidine-containing dipeptides and metabolites assessed in the *Pectoralis major* muscles from broiler chickens fed diets low in lysine during different feeding phases: grower I (GRW I) or grower I and II (GRW I + II). *n* = 6 samples/group; sem = standard error of mean; ns = not significant.

	Experimental Group			sem	*p*-Value	
	CONT	GRW I	GRW I + II		Analysis of Variance ^1^	Planned Contrast ^2^
Histidine-containing dipeptides (mg/100 g of meat)						
Anserine	369.7	429.4	442.3	15.5	ns	*
Carnosine	108.2	123.3	139.5	8.4	ns	ns
Metabolites (mg/100 g of meat)						
Creatine	330.2	363.3	345.7	7.4	ns	ns
IMP	114.9	125.1	117.8	4.4	ns	ns
Lactate	611.1	692.9	677.5	18.4	ns	0.06
Fumarate	0.99	0.98	0.89	0.03	ns	ns
Hypoxanthine	23.4	27.6	27.1	1.1	ns	0.09
Guanidoacetate	29.8	30.6	30.4	0.4	ns	ns
Glucose	14.3	22.5	19.4	2.1	ns	ns
Inosine	34.9	32.8	29.5	1.7	ns	ns

^1^ One-way ANOVA considering the lysine restriction implemented during different feeding phases as a main effect. ^2^ Planned orthogonal contrast: Control (CONT) vs. Treated (GRW I and GRW I + II) groups. * = *p* < 0.05; ns = not significant.

**Table 7:** Buffering capacity of the *Pectoralis major* muscles from broiler chickens fed diets low in lysine during different feeding phases: grower I (GRW I) or grower I and II (GRW I + II). Values are expressed as µmol H ^+^·pH^−1^·g^−1^. *n* = 6 samples/group; sem = standard error of mean; ns = not significant.

	Experimental Group			sem	*p*-Value	
	CONT	GRW I	GRW I + II		Analysis of Variance ^1^	Planned Contrast ^2^
pH 6.0	41.2	42.5	44.2	0.9	ns	ns
pH 6.2	44.4	42.3	51.5	1.3	ns	ns
pH 6.4	48.6	46.8	47.7	1.0	ns	ns
pH 6.6	49.2 ab	45.6 b	51.6 a	1.3	**	ns
pH 6.8	49.5 b	44.0 b	58.0 a	1.4	*	ns
pH 7.0	44.5 b	42.1 b	55.2 a	1.4	**	ns

^1^ One-way ANOVA considering lysine restriction implemented during different feeding phases as a main effect. ^2^ Planned orthogonal contrast: Control (CONT) vs. Treated (GRW I and GRW I + II) groups. * = *p* < 0.05; ** = *p* ≤ 0.01; ns = not significant.

## Data Availability

The data presented in this study are available on request from the corresponding author.
